# Associations of high-sensitivity C-reactive protein with neuropsychological outcomes and cerebral white matter hyperintensities in older adults at risk of dementia

**DOI:** 10.1016/j.bbih.2024.100924

**Published:** 2024-12-11

**Authors:** Rachael Yu, Shawn D. Kong, Catriona Ireland, Genevieve Z. Steiner-Lim, Kimberley Bassett, Hannes Almgren, Dongang Wang, Chenyu Wang, Johannes C. Michaelian, Sharon L. Naismith

**Affiliations:** a Healthy Brain Ageing Program, Brain and Mind Centre, School of Psychology, Faculty of Science, University of Sydney, NSW, 2050, Australia; b Charles Perkins Centre, University of Sydney, Camperdown, NSW, 2050, Australia; c NICM Health Research Institute, Western Sydney University, Australia; d School of Biomedical Engineering, Faculty of Engineering, The University of Sydney, NSW, Australia; e Sydney Neuroimaging Analysis Centre, Brain and Mind Centre, University of Sydney, NSW, 2050, Australia

**Keywords:** Inflammation, C-reactive protein, Dementia, Subjective cognitive decline (SCD), Mild cognitive impairment (MCI), White matter hyperintensities (WMH)

## Abstract

Inflammation is becoming increasingly recognised as a core feature of dementia with evidence indicating that its role may vary and adapt across different stages of the neurodegenerative process. This study aimed to investigate whether the associations of high-sensitivity C-reactive protein (hs-CRP) with neuropsychological performance (verbal memory, executive function, processing speed) and cerebral white matter hyperintensities (WMHs) differed between older adults with subjective cognitive decline (SCD; *n* = 179) and mild cognitive impairment (MCI; *n* = 286). Fasting serum hs-CRP concentrations were grouped into low (<1.0 mg/L), moderate (1.0–3.0 mg/L), and high (>3.0–10.0 mg/L). Structural MRI scans were used to estimate WMH lesion volumes in the whole brain, as well as periventricular, deep white matter, and frontal regions. After adjusting for relevant demographic and clinical factors, multiple regression analyses revealed that in participants with SCD, high hs-CRP concentrations were significantly associated with poorer executive function (β[95% CI] = −.20[−.65, −.04], *p* = .025) and processing speed (β[95% CI] = −.19[−.53, .00], *p* = .048). Exploratory analyses suggested that this effect may be specific to APOE-ε4 non-carriers only. There were no significant associations between hs-CRP and neuropsychological outcomes in those with MCI. Hs-CRP was not associated with WMH volumes. Our findings suggest that hs-CRP may be involved in early disruptions to cerebral frontal-subcortical pathways, particularly in APOE-ε4 non-carriers, though this association may be independent of white matter lesions. In the earliest stages of cognitive decline where subjective complaints are paramount, addressing inflammation may offer potential benefits for supporting cognitive health.

## Highlights

• Elevated C-reactive protein linked to poorer executive function and processing speed.
• Seen in subjective cognitive decline (SCD) but not mild cognitive impairment (MCI).
• Independent of white matter hyperintensities and other risk factors of dementia.

## 1 Introduction

With the global increase in life expectancy, a sharp rise in the prevalence of age-related neurodegenerative diseases such as dementia is expected, projected to double from a current estimate of 55 million people to 116 million by 2050 (Gustavsson et al., 2023). The most common forms of dementia are Alzheimer's disease (AD) and vascular dementia (VaD), accounting for up to 70% and 20% of all cases respectively, though the majority of cases present with a mixed aetiology of both AD and VaD pathologies (Akhter et al., 2021; Attems and Jellinger, 2014). Subjective cognitive decline (SCD), which is the self-reported experience of cognitive difficulties in the absence of objective impairment, is considered to be a risk factor for dementia and potentially the earliest clinical manifestation (Jessen et al., 2014). Mild cognitive impairment (MCI) is a classification used for individuals exhibiting objectively impaired cognitive function during neuropsychological testing, but that of which does not significantly interfere with daily functioning (Winblad et al., 2004). It is estimated that approximately 40% of those with MCI will progress to dementia within five years (Mitchell and Shiri-Feshki, 2009). As such, SCD and MCI are considered ‘at-risk’ stages of dementia, where it is particularly crucial to elucidate early pathophysiological mechanisms that occur in these key periods, in order to inform primary and secondary prevention strategies.

Mounting evidence in recent years has linked inflammation with cognitive decline and an increased risk of dementia, through pathways such as blood-brain barrier disruption, exacerbation of neurodegenerative pathologies (e.g. β-amyloid [Aβ] and hyperphosphorylated tau in AD), and cerebrovascular damage (Ahmad et al., 2022; Walker et al., 2023). C-reactive protein (CRP), along with its high-sensitivity assay (hs-CRP), is a downstream acute-phase reactant marker synthesised in the liver in response to inflammatory reactions as part of the innate immune response (Banait et al., 2022). Higher CRP levels have been linked to poorer global cognitive performance in healthy older adults (Lewis and Knight, 2021; Wang et al., 2023; Yaffe et al., 2003), and are associated with an increased risk of developing dementia 25 years later (Schmidt et al., 2002). In a recent study, the relationship between hs-CRP concentration and performance on a global measure of cognitive function was observed to vary across different stages of dementia progression (Zhang et al., 2023). Elevated hs-CRP levels showed a trend towards poorer cognition in individuals experiencing SCD, though this did not reach statistical significance (Zhang et al., 2023). No association was found between hs-CRP and cognition in individuals with MCI (Zhang et al., 2023). Interestingly, higher concentrations of CRP are associated with a slowing of cognitive decline in patients diagnosed with AD (Lewis and Knight, 2021; Zhang et al., 2023). These findings suggest a complex and disease stage-dependent relationship between inflammation and cognitive function.

Existing literature on the relationship between CRP and cognition has predominantly relied on screening tests of global cognition (e.g. Feng et al., 2023), which provide a generalised and non-specific overview of the patient's cognitive status. However, general screening tests may be less sensitive in detecting subtle cognitive changes, particularly in early stages of cognitive decline. Further, understanding how CRP may be associated with domain-specific cognitive outcomes could provide novel insights into preferential brain regions and circuitry affected by inflammation. Some evidence suggests that high CRP levels are linked with poorer performance in domains mediated by frontal-subcortical pathways (e.g. attention, executive function and processing speed) in cognitively healthy, community-dwelling older adults (Tegeler et al., 2016; Wersching et al., 2010). However, there is now a need to explore the full spectrum of preclinical, or ‘at-risk’, stages of dementia to determine the links between inflammation and cognition throughout disease progression.

Impairments in cognitive functions such as executive function and processing speed often result from disruptions of white matter fibre tracts throughout the brain (Alber et al., 2019), suggesting a potential pathway in which elevated levels of CRP may affect these neuropsychological outcomes. White matter hyperintensities (WMHs), visualised on magnetic resonance imaging (MRI), reflect pathological white matter changes including damaged myelination and ischemic lesions, and serve as a marker of small vessel disease, which is considered a significant risk factor for AD and VaD (Alber et al., 2019; Lee et al., 2016). However, conflicting evidence exists regarding the association between CRP levels and WMH volume with some studies suggesting a positive association in older adults (Satizabal et al., 2012; van Dijk et al., 2005; Wright et al., 2009), while others do not (Boots et al., 2020; Wersching et al., 2010). Discrepancies in previous findings may be due to variations in sample characteristics (e.g. community-dwelling vs. clinical cohorts) or investigations of total WMH volume vs. regional analyses. Notably, WMHs located in the periventricular and deep white matter regions are linked to executive dysfunction, possibly due to disrupted connections with frontal circuits (Bolandzadeh et al., 2012).

Existing statistical analyses in studies have typically adjusted for basic demographic variables such as age, sex, and education. Beyond the well-known genetic risk factor for AD, the presence of the Apolipoprotein E (*APOE*) ε4 allele, several lifestyle and acquired clinical conditions are recognised as modifiable risk factors for dementia, many of which are associated with elevated inflammatory processes. These include cardiovascular risk burden (e.g. high blood pressure and cholesterol, diabetes, and smoking), depression, sleep quality, and alcohol consumption, all of which have independent relationships with inflammation and cognition (Allison and Ditor, 2014; Jia et al., 2023; Jin et al., 2023; Wang et al., 2010) and white matter disease (Daviet et al., 2022; He et al., 2022; Hidese et al., 2023; Youssef et al., 2023). Therefore, it remains unclear whether CRP is associated with cognitive and white matter outcomes beyond the influence of these modifiable risk factors.

To date, studies examining the impact of inflammation on cognitive outcomes have primarily focused on individuals at various stages of preclinical dementia using a global measure of cognition, or examined specific neuropsychological domains in homogeneous samples (i.e., cognitively healthy individuals). To this end, the present study aimed to bridge this gap by exploring the relationship between hs-CRP levels and performance across different neuropsychological domains (verbal memory, executive function, processing speed), and determining how these associations may vary across dementia risk stages (i.e., SCD and MCI). Consistent with current knowledge, we hypothesised that elevated peripheral hs-CRP concentrations would be significantly associated with performance in executive function and processing speed in the SCD group but not in individuals with MCI. Further, we aimed to elucidate the relationship between hs-CRP and WMH volumes, particularly in the periventricular, deep white matter, and frontal regions of the brain. Finally, we sought to determine whether such associations persisted after adjusting for established modifiable dementia risk factors.

## 2 Methods

### 2.1 Participants

Participants aged ≥50 years were recruited from the Healthy Brain Ageing (HBA) Clinic, a specialist early-intervention memory and cognition clinic in Sydney, Australia, for older adults referred by a physician due to experiencing self- or carer-reported new-onset cognitive concerns. Exclusion criteria for participation at the HBA clinic include history of stroke and head injury with loss of consciousness >30min, neurological disease (e.g. Parkinson's, epilepsy) or non-affective psychiatric illness (e.g. attention-deficit/hyperactivity disorder, schizophrenia), intellectual disability, insufficient English for neuropsychological assessment, excessive alcohol consumption (>14 standard drinks per week), or existing diagnosis of dementia and/or MMSE <20. For this study, only participants with hs-CRP data available were included. All participants provided written consent prior to study participation, and all research activities were approved by the University of Sydney Human Research Ethics Committee (Protocol numbers: 2012/1873 and 2019/HE000271). All study methods were conducted in compliance with the Helsinki Declaration.

### 2.2 Clinical assessments

#### 2.2.1 Neuropsychological assessment

A comprehensive, standardised neuropsychological assessment was conducted by a clinical neuropsychologist. Raw scores were converted into *z*-scores adjusted for age and education attainment. Composite scores were calculated as an average of *z*-scores of tests within the same cognitive domain.

• *Verbal Memory –* Rey Auditory Verbal Learning Test (delayed recall score) (Schmidt, 1996) and the Logical Memory II subtest of the Wechsler Memory Scale-III (delayed recall) (Wechsler, 1997);
• *Executive Function* – Trail Making Test part B (Reitan, 1959), Colour Word Interference Test (CWIT; Delis-Kaplan Executive Function System) Condition 3 and Condition 4 (Delis et al., 2001), Digit Span (total score) (Wechsler, 1997) and F-A-S Verbal Fluency Test (Tombaugh et al., 1999);
• *Processing speed* – Trail Making Test part A (Reitan, 1959), and CWIT Condition 1 and Condition 2 (Delis et al., 2001).

Composite scores were not calculated if one or more tests were missing for any participant. The Wechsler Test of Adult Reading – Full Scale IQ (WTAR-FSIQ) was used to estimate premorbid intellectual ability for descriptive purposes (Wechsler, 2001).

Classifications of MCI were determined by clinician concordance, using the Winblad clinical criteria of obtaining at least 1.5 SDs below age- and education-adjusted normative scores on neuropsychological assessments (Winblad et al., 2004). Participants who did not meet criteria for MCI were classified as experiencing SCD.

#### 2.2.2 C-reactive protein

All participants were provided a referral for a fasting clinical blood test at a commercial Australian pathology collection centre (Douglass Hanly Moir, AU). Hs-CRP was grouped into three categories as defined by the US Centres for Disease Control and Prevention: low <1.0 mg/L, intermediate 1.0–3.0 mg/L, and high >3.0 mg/L (Pearson et al., 2003). Participants with hs-CRP concentrations more than 10 mg/L were excluded due to suggestive evidence of an acute infection (Pearson et al., 2003).

#### 2.2.3 MRI acquisition and processing

Participants completed the MRI protocol within four weeks of the clinical assessment at two facilities in Sydney, Australia. For site 1, the structural MRI data were collected using a 3-T General Electric (GE) Discovery MR750 scanner (GE Medical Systems, USA). 39.3% (*n* = 110) of our MRI subsample was collected using an 8-channel phased-array head coil with the following T1 weighted imaging sequence: 3D-T1-weighted BRAVO Spoiled Gradient-Recalled (SPGR) sequence, acquiring 196 sagittal slices (repetition time = 7.2 ms; echo time = 2.8 ms; flip angle = 12; matrix 256 × 256; .9 mm isotropic voxels). Further, the following sagittal 3D FLAIR sequence was used: repetition time = 8000ms; echo time = 160ms; inversion time = 2100ms; flip angle = 90.0; matrix = 256 × 224; voxel size = 0.9 × 1.1 × 1.2 mm; 1.2 mm slices. The structural MRI data for 113 participants (40.4%) were performed on a 32-channel phased-array head coil. Again, a 3D T1-weighted BRAVO Spoiled Gradient-Recalled (SPGR) sequence was used (176 sagittal slices; repetition time = 7.4ms; echo time = 3.0ms; flip angle = 11; matrix 256 × 256; 1.0 mm isotropic voxels). Sagittal 3D FLAIR images were also collected during this period (repetition time = 7000ms; echo time = 159ms; inversion time = 1860ms; matrix = 256 × 256; voxel size = 1.0 × 1.0 × 1.0 mm; 1.0 mm slices). For site 2 (*n* = 57, 20.4%), a 3-T Siemens Magnetom Skyra scanner (SIEMENS, Erlangen, Germany) with a 20-channel phased-array head coil was used. The scan included a T1 weighted MPRAGE imaging sequence (208 sagittal slices; repetition time = 2200ms; echo time = 2.48ms; flip angle = 8.0; matrix 232 × 256; .9 mm isotropic voxels). Sagittal 3D FLAIR images were also acquired (repetition time = 7000ms; echo time = 383ms; inversion time = 2100ms; flip angle = 120; matrix = 256 × 199; voxel size = 1.28 × 1.00 × 1.29 mm; 1.29 mm slices).

Sagittal 3D FLAIR scans (either 1 mm isotropic or 1 × 0.5 × 0.5 mm resolutions), and 3D T1-weighted scans (1 mm isotropic) were used to estimate WMH lesion volumes. Estimations were automatically calculated using iQ-Solutions, an FDA 510(k) cleared AI-based MRI analysis tool (Barnett et al., 2023). Lesion masks were automatically generated from FLAIR images, which were extracted and co-registered to the T1-weighted image (FLIRT; FSL, version 6.0.0, [38]). The estimation of lesion volumes in subgroups are conducted based on the outputs of iQ-solutions. Automatically segmented lesions were mapped to corresponding periventricular, deep white matter, and frontal lobes masks by nearest neighbour algorithm. The standard templates were provided by FSL (periventricular and deep white matter) and iQ-solutions (lobes). Each lesion was considered separately, and any lesion that sat in the boundaries of two different regions were assigned to the region that contained the majority of volumes of that lesion.

#### 2.2.4 APOE genotyping

DNA for genotyping was extracted from peripheral blood mononuclear cells separated from centrifuged plasma-blood samples collected at the time of clinical assessment for 300 participants (64.5% of total sample). *APOE* genotyping was performed using the UK Biobank Axiom™ Array at the Ramaciotti Centre for Genomics, UNSW Australia (NATA ISO/IEC Accreditation Number #20417) probing single nucleotide polymorphisms (SNPs) rs429358 and rs7412 in the *APOE* gene. Subsequently, APOE-ε4 status was categorized as a binary variable, distinguishing between individuals who do not carry the ε4 allele (non-carriers) and those who possess at least one ε4 allele (carriers).

#### 2.2.5 Other covariates

A priori covariates were measured in addition to demographic variables (age, sex, and years of education). A semi-structured full medical assessment was conducted by a physician, which included the MMSE, anthropometric measurements (including body mass index [BMI] and blood pressure), and a detailed medical and lifestyle history (including alcohol consumption). Total disease burden was measured using the Cumulative Illness Rating Scale-Geriatric version (CIRS-G), a comprehensive, multidimensional scale assessing illness burden across 13 organ systems (Miller et al., 1992). Participants completed the Pittsburgh Sleep Quality Index (PSQI; Buysse et al., 1989) and Geriatric Depression Scale-15 item questionnaire (GDS-15; Almeida and Almeida, 1999) to assess self-reported sleep quality and depressive symptoms respectively. The Framingham General Cardiovascular Risk Score (FGCRS) was derived from the participant's age, sex, high-density lipoprotein, total cholesterol, systolic blood pressure, hypertension treatment, current smoking status, and diabetes diagnosis (D'Agostino et al., 2008). Diabetes diagnosis was determined as fasting blood glucose ≥7.0 mmol/L, HbA1c level ≥47.5 mmol/mol (6.5%) and/or self-reported diagnosed diabetes.

### 2.3 Statistical analysis

All statistical analyses were conducted on SPSS Version 28 (SPSS Inc., IBM Corp. In Armonk, NY, USA). Missing values for covariates (17.2% for PSQI total score, max 8.7% for all other covariates) were estimated using multiple imputation analysis based on linear regression. Due to only 64.5% of our sample having *APOE* genotyping data, all analyses that included APOE-e4 carrier status were conducted in this subsample (*n* = 300).

Differences in demographic, clinical and neuroimaging variables between the SCD and MCI groups were investigated using the chi-squared test for categorical variables, independent t-tests for normally distributed variables and Mann-Whitney U tests for non-normally distributed variables. Correlations at the univariate level were conducted using Pearson's correlations for normally distributed data, and Kendall's tau-b correlations for non-normally distributed data. Binary variables were coded as follows: Sex (Male = 0; Female = 1); APOE-ε4 (Non-carrier = 0, Carrier = 1).

A series of multivariate linear regression analyses were conducted to evaluate the contributions of hs-CRP to each neuropsychological and white matter outcome, for SCD and MCI groups separately. Hs-CRP categories were dummy coded to compare moderate and high categories to the low group. WMH volumes for total brain and each region were log-transformed to normalise distributions. In Model 1, non-modifiable (demographic) variables of age, sex, education, and total disease burden (CIRS-G total score) were included as covariates. Analyses of WMH outcomes further adjusted for scan sequence type (8, 32 or 20-channel phased-array head coil). APOE-ε4 carrier status was also included in Model 1 as a covariate for the cognitive outcomes in the sub-sample where genotyping data was available. In Model 2, we examined whether hs-CRP would predict outcomes beyond the contributions of modifiable risk factors determined a priori, by additionally including depressive symptoms (GDS-15), alcohol consumption, BMI, cardiovascular health (FGCRS) and sleep quality (PSQI total score) into the model. Standardised beta coefficients with 95% confidence intervals were calculated as estimates of effect size.

## 3 Results

### 3.1 Sample characteristics

16 participants had hs-CRP concentrations of >10 mg/L and were excluded (8 with SCD and 8 with MCI). The final sample consisted of 465 participants (Mean age = 67.7 years [SD = 8.1], 62.8% female, median MMSE = 29.0 [IQR = 2]), who were classified as either having SCD (*n* = 179, 38.5%) or MCI (*n* = 286, 61.5%). Across the full sample, 42 participants (9%) were missing at least one neuropsychological outcome due to missing one or more tests used in the calculation of the composite score. Those with missing neuropsychological data did not differ with the rest of the sample with regards to age, sex, MMSE or diagnostic classification.

See Table 1a for demographic characteristics and neuropsychological test scores for the total, SCD and MCI samples. Overall, the sample was well educated, with an average of 14.4 years of education (SD = 3.0), and mean estimated IQ in the average range (106.5, SD = 8.2). Depressive symptoms across the sample were within the normal range (median GDS-15 = 3.0; 14.2% currently meeting DSM-IV criteria for Major Depressive Disorder) with only 22.8% currently taking antidepressants. The sample had low medical burden (mean CIRS-G = 5.9) but reported slightly poor sleep quality on average (mean PSQI total score = 6.9). 37.7% of participants carried at least one APOE-ε4 allele.

**Table 1a:** Sample characteristics, overall and stratified by diagnostic classification.

	Total Sample (*n* = 465)		SCD (*n* = 179)		MCI (*n* = 286)		
Variable	Mean/median	SD/IQR	Mean/median	SD/IQR	Mean/median	SD/IQR	*p*
**Descriptive**							
Age^a^	67.65	8.09	66.46	7.65	68.40	8.29	**.012**
Sex, female^b^	292 (62.8%)		120 (67.0%)		172 (60.1%)		.134
Education (years)^a^	14.44	3.00	14.20	2.79	14.59	3.10	.165
WTAR FSIQ^a^	106.50	8.18	106.07	8.03	106.77	8.28	.375
**Cognitive**							
Verbal Memory^a^,^d^	.18	1.01	.80	.63	−.21	1.02	**<.001**
Executive Function^a^,^d^	.26	.67	.56	.54	.08	.69	**<.001**
Processing Speed^a^,^d^	.20	.68	.50	.46	.03	.73	**<.001**
MMSE^c^	29.00	28.0–30.0	30.00	29.0–30.0	29.00	28.0–30.0	**<.001**
**Clinical**							
CIRS-G total score^a^	5.95	3.54	5.83	3.49	6.02	3.58	.561
Current antidepressant use^b^	106 (22.8%)		43 (24.0%)		63 (22.0%)		.618
Current depression^b^	66 (14.2%)		23 (12.8%)		43 (15.0%)		.511
GDS-15^c^	3.00	1.0–6.0	3.00	1.0–5.1	3.00	1.0–6.0	.506
Alcohol (drinks per week)^c^	2.00	.0–7.25	2.00	.0–9.0	1.0	.0–7.0	**.032**
BMI^a^	26.90	4.79	26.78	4.97	26.99	4.68	.646
FGCRS^a^	14.40	3.76	13.77	3.71	14.79	3.74	**.004**
PSQI total score^a^	6.92	3.45	7.37	3.56	6.63	3.35	**.025**
**Biochemistry analysis**							
Hs-CRP (mg/L)^c^	1.00	.58–2.00	1.00	.5–2.0	1.00	.6–2.0	.718
Hs-CRP group^b^							.493
*Low*	221 (47.5%)		81 (45.3%)		140 (49.0%)		
*Moderate*	187 (40.2%)		78 (43.6%)		109 (38.1%)		
*High*	57 (12.3%)		20 (11.2%)		37 (12.9%)		

**Genotyping**	**Total sample** (*n* = 300)		**SCD** (*n* = 133)		**MCI** (*n* = 167)		
*APOE-*ε4 carrier^b^	113 (37.7%)		46 (34.6%)		67 (40.1%)		.326

Note. SCD = Subjective cognitive decline; MCI = Mild cognitive impairment; SD = standard deviation; IQR = interquartile range; WTAR FSIQ = Wechsler Test of Adult Reading – Full scale IQ; MMSE = Mini-Mental State Examination; CIRS-G = Cumulative Illness Rating Scale-Geriatric version; GDS-15 = Geriatric Depression Scale-15 item; BMI = Body mass index; FGCRS = Framingham General Cardiovascular Risk Score; PSQI = Pittsburgh Sleep Quality Index; Hs-CRP = High sensitivity C-reactive protein; APOE = Apolipoprotein E.

a Mean, SD and independent samples T-test.

b Frequency and Chi-square test.

c Median, interquartile range and Mann-Whitney *U* test.

d For the outcome variables, 4 participants were missing data for Verbal Memory (1 with SCD, 3 with MCI); 38 participants were missing data for Executive Function (20 with SCD, 18 with MCI); 25 participants were missing data for Processing Speed (16 with SCD, 9 with MCI).

As expected, participants with MCI were significantly older, and had poorer test scores for the MMSE and all neuropsychological domains, compared to those with SCD (*p* < .001). Participants with MCI additionally had poorer cardiovascular health (FGCRS), but lower alcohol consumption and better sleep quality, compared to participants with SCD. Hs-CRP concentrations did not differ between those with SCD and MCI (*p* > .05).

A subsample of 281 participants had MRI data (see Table 1b). One participant was excluded due to an error in segmentation of WMH volumes. The final subsample (*N* = 280; SCD, *n* = 109; MCI, *n* = 171) did not differ from the main sample in regards to age, sex, MMSE, diagnostic classification or hs-CRP concentrations. Compared to those with SCD, participants with MCI had significantly increased number and volume of WMH lesions in the whole brain, deep white matter, and frontal lobe. Those with MCI additionally had significantly increased volume of WMHs in the periventricular region.

**Table 1b:** White matter hyperintensities in neuroimaging subsample.

	Total Sample (*n* = 280)		SCD (*n* = 109)		MCI (*n* = 171)		
**Variable**	**Mean/Median**	**SD/IQR**	**Mean/Median**	**SD/IQR**	**Mean/Median**	**SD/IQR**	*p*
**Whole brain**							
Volume (mm^3^)^a^	1282.80	296.22–4869.20	932.58	231.65–2656.34	1618.97	400.94–6222.56	**.007**
Volume (log-transformed)^b^	7.15	1.71	6.80	1.62	7.37	1.74	**.006**
Lesions^a^	34.00	16.25–69.75	26.00	11.50–57.00	43.00	19.00–75.00	**.003**
**Periventricular**							
Volume (mm^3^)^a^	549.60	74.49–3269.81	446.08	58.61–1188.15	809.05	82.64–4131.47	**.046**
Volume (log-transformed)^b^	5.93	2.72	5.60	2.49	6.14	2.84	.106
Lesions^a^	6.00	3.00–10.00	6.00	2.00–9.00	6.00	3.00–11.00	.123
**Deep white matter**							
Volume (mm^3^)^a^	305.06	113.54–706.78	243.41	83.86–603.23	351.88	136.41–831.25	**.008**
Volume (log-transformed)^b^	5.65	1.31	5.35	1.35	5.84	1.25	**.002**
Lesions^a^	17.50	9.00–38.00	13.00	6.00–32.50	21.00	10.00–45.00	**<.001**
**Frontal lobe**							
Volume (mm^3^)^a^	773.86	159.58–3103.84	513.56	107.47–1686.47	943.67	203.87–3479.40	**.002**
Volume (log-transformed)^b^	6.42	2.04	5.98	2.00	6.69	2.02	**.004**
Lesions^a^	17.00	8.00–36.75	11.00	6.00–31.00	23.00	10.00–41.00	**.001**

Note. SCD = Subjective cognitive decline; MCI = Mild cognitive impairment; SD = standard deviation; IQR = interquartile range.

a Median, interquartile range and Mann-Whitney *U* test.

b Mean, SD and independent samples T-test.

### 3.2 Correlations of hs-CRP, neuropsychological outcomes, and white matter hyperintensities

See Table 2 for unadjusted correlates of hs-CRP with descriptive, clinical, cognitive, and white matter outcomes, for SCD and MCI groups. APOE-ε4 was associated with lower hs-CRP concentrations in the MCI group. Higher hs-CRP levels were significantly correlated with poorer performance in executive function for individuals with SCD, but not for participants with MCI. Additionally, in those with SCD, higher hs-CRP concentrations were significantly correlated with increased WMH volume in deep white matter.

**Table 2:** Correlates of high-sensitivity C-reactive protein (log-transformed), stratified by diagnostic classification.

Hs-CRP		
**Variable**	**SCD**	**MCI**
**Descriptive**		
Age^a^	.040	.058
Sex^b^	.175∗∗	.015
Education (years)^a^	−.096	−.086
**Clinical**		
APOE-ε4 carrier^b^	.030	−.170∗∗
CIRS-G total score^a^	.183∗	.212∗∗
GDS-15^b^	−.016	−.005
Alcohol (drinks per week)^b^	−.052	−.032
BMI^a^	.426∗∗	.449∗∗
FGCRS^a^	.201∗∗	.083
PSQI total score^a^	−.005	−.052
**Cognitive**		
MMSE^b^	−.072	−.047
Verbal Memory^a^	.013	.013
Executive Function^a^	−.196∗	−.059
Processing Speed^a^	−.071	−.022
**WMH volume**^a^		
Whole brain	.104	−.010
Periventricular	.129	−.013
Deep white matter	.200∗	−.061
Frontal lobe	.100	.031

Note. Hs-CRP and WMH volumes were log-transformed. Hs-CRP = high-sensitivity C-reactive protein; SCD = Subjective cognitive decline; MCI = Mild cognitive impairment; APOE = Apolipoprotein E; CIRS-G = Cumulative Illness Rating Scale-Geriatric version; GDS-15 = Geriatric Depression Scale-15 item; BMI = Body mass index; FGCRS = Framingham General Cardiovascular Risk Score; PSQI = Pittsburgh Sleep Quality Index; MMSE = Mini-mental state examination; WMH = white matter hyperintensities.

∗p < .05.

∗∗p < .01.

a Pearson's Correlation.

b Kendall's tau-b Correlation.

Unadjusted correlations of neuropsychological performance with covariates and white matter outcomes are summarised in Table 3. Higher WMH volumes located in the periventricular and frontal lobe regions were significantly correlated with poorer processing speed performance for both SCD and MCI groups. Higher WMH volumes in the whole brain, deep white matter, and frontal lobe were significantly correlated with poorer executive functioning in those with MCI (but not SCD).

**Table 3:** Factors associated with neuropsychological performance, stratified by diagnostic classification.

	Verbal Memory		Executive Function		Processing Speed	
	SCD	MCI	SCD	MCI	SCD	MCI
**Descriptive**						
Age^a^	−.026	−.073	.001	.042	−.060	−.073
Sex^b^	.187∗∗	.155∗∗	−.014	.008	−.088	.021
Education (years)^a^	.267∗∗	.167∗∗	.210∗∗	.238∗∗	.152	.146∗
**Clinical**						
APOE-ε4 carrier^b^	.070	−.081	−.048	−.035	−.086	−.079
CIRS-G total score^a^	−.050	.063	−.120	−.059	−.157∗	−.073
GDS-15^b^	−.028	−.016	.075	−.110∗	.040	−.036
Alcohol (drinks per week)^b^	.028	.095∗	.141∗	.059	.079	.032
BMI^a^	−.192∗	−.026	−.158∗	−.177∗∗	.024	−.061
FGCRS^a^	−.198∗	−.025	.057	−.095	.076	−.098
PSQI total score^a^	−.101	.088	.001	−.141∗	−.058	.004
**WMH volume**^a^,^c^						
Whole brain	.000	−.044	−.004	−.195∗	−.146	−.183∗
Periventricular	−.041	−.061	−.029	−.149	−.218∗	−.206∗∗
Deep white matter	−.026	.064	.002	−.183∗	.032	−.093
Frontal lobe	−.043	−.034	−.030	−.190∗	−.226∗	−.195∗

Note. SCD = Subjective cognitive decline; MCI = Mild cognitive impairment; APOE = Apolipoprotein E; CIRS-G = Cumulative Illness Rating Scale-Geriatric version; GDS-15 = Geriatric Depression Scale-15 item; BMI = Body mass index; FGCRS = Framingham General Cardiovascular Risk Score; PSQI = Pittsburgh Sleep Quality Index; WMH = white matter hyperintensities.

∗p < .05.

∗∗p < .01.

a Pearson's Correlation.

b Kendall's tau-b Correlation.

c Log transformed.

### 3.3 Hs-CRP as a predictor of neuropsychological outcomes and white matter outcomes

All assumptions for multiple linear regression were met. In the SCD group, high hs-CRP concentrations were significantly associated with poorer executive function after adjusting for demographic variables (Model 1; β[95% CI] = −.20[−.62, −.05], *p* = .022), and this result persisted after controlling for additional modifiable risk factors (Model 2; β[95% CI] = −.20[−.65, −.04], *p* = .025). The effect of high hs-CRP on executive function remained after controlling for APOE-ε4 status (Model 1: β[95% CI] = −.25[−.82, −.11], *p* = .012; Model 2: β[95% CI] = −.26[−.87, −.07], *p* = .021).

Additionally, in those with SCD, high hs-CRP was significantly associated with poorer processing speed after adjustment of a priori covariates (Model 2; β[95% CI] = −.19[−.53, .00], *p* = .048). This effect remained after controlling for APOE-ε4 status (Model 2: β[95% CI] = −.25[−.74, −.04], *p* = .031).

**Fig. 1:**
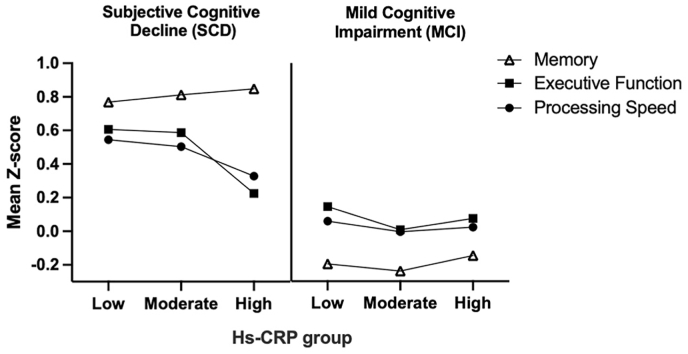
Associations between hs-CRP and neuropsychological outcomes in SCD and MCI groups. Note. Hs-CRP = high-sensitivity C-reactive protein. Groupings of hs-CRP concentrations are defined by the US Centres for Disease Control and Prevention; low (<1.0 mg/L), moderate (1.0–3.0 mg/L), and high (>3.0–10.0 mg/L).

Hs-CRP was not associated with neuropsychological outcomes for individuals with MCI (see Fig. 1). See Supplementary Table S1 for results of multivariate linear regressions.

There were no significant associations between hs-CRP and WMH volume in any of the regions (total, periventricular, deep white matter, and frontal lobe) in the SCD or MCI groups after adjustment of demographic variables (Model 1) or additional risk factors (Model 2), *p*-values ≥.061 (see Supplementary Table S2).

### 3.4 Exploratory analyses stratified by APOE status

We further analysed the contributions of hs-CRP on cognitive outcomes stratified by APOE-ε4 status, adjusted for age, sex, education and CIRS-G. High hs-CRP was associated with poorer executive function in APOE-ε4 non-carriers (β[95% CI] = −.20[−.74, −.08], *p* = .016, *n* = 187) but not in ε4 carriers (β[95% CI] = −.02[−.39, .49], *p* = .813, *n* = 113). Similarly, high hs-CRP concentrations trended towards poorer processing speed in APOE-ε4 non-carriers (β[95% CI] = −.16[−.63, .01], *p* = .059) but not in ε4 carriers (β[95% CI] = −.02[−.52, .41], *p* = .822).

Furthermore, adjusted multiple regression stratified for both diagnostic classification and APOE-ε4 status showed that the effect of high hs-CRP on poorer executive function was specific to APOE-ε4 non-carriers with SCD (β[95% CI] = −.31[−1.06, −.12], *p* = .014, *n* = 87). Similar trends were observed on the effects of high hs-CRP on processing speed in APOE-ε4 non-carriers with SCD (β[95% CI] = −.23[−.77, .02], *p* = .063). Of note, high hs-CRP levels trended towards better verbal memory performance in APOE-ε4 carriers with MCI (β[95% CI] = .25[−.05, 1.83], *p* = .064, *n* = 67). Hs-CRP was not associated with any other neuropsychological outcomes for APOE-ε4 carriers or individuals with MCI.

## 4 Discussion

This cross-sectional study aimed to understand the relationship between hs-CRP and neuropsychological performance across multiple domains in older adults at risk of dementia (i.e., SCD and MCI). After controlling for known modifiable risk factors of dementia, higher concentrations of hs-CRP were associated with poorer executive function and processing speed but only in the SCD stage, and not in those with MCI. Exploratory analyses suggested that this effect was driven by APOE-ε4 non-carriers. Findings also show that while individuals with MCI had increased WMH volumes and lesions compared to those with SCD, hs-CRP was not independently associated with WMH volumes beyond the effects of basic demographic factors.

Our findings align with prior work suggesting a harmful role of hs-CRP in early cognitive disruption (i.e., SCD) but not in later stages of objective cognitive decline (i.e., in MCI; Zhang et al., 2023). However, we extend this understanding to apply to the more distinct neuropsychological domains of executive functioning and processing speed, with the latter statistically significant in the presence of modifiable risk factors (Model 2). Reactive inflammatory processes in response to emerging pathologies in the brain are suggested to interact with, and accelerate, the neurodegenerative processes, which may further explain observations that high baseline CRP levels in cognitively healthy individuals increase the risk of developing dementia in later years (Lewis and Knight, 2021; Schmidt et al., 2002).

It is important to note that, while statistically significant, we only found moderate associations between hs-CRP and cognitive function. This suggests that while elevated circulating CRP may be involved in the physiological processes associated with poorer cognition, even the high-sensitivity assay may not be clinically suitable as a marker for cognitive decline (Tegeler et al., 2016). Nevertheless, our results provide support for the notion that targeting inflammation at this earliest stage of cognitive concern may support cognitive health, particularly in manifestations of executive dysfunction. While “upstream” markers (e.g. IL-6, IL-1β, TNFα) and markers of astrocytic and microglial activation (e.g. YKL-40, sTREM2) may better reflect neuroinflammatory processes (Nordengen et al., 2019; Kwon and Koh, 2020), our findings highlight the need for future replication and longitudinal investigations to focus on how the roles of these inflammatory mediators may evolve across stages of cognitive status and disease progression.

Contrarily to cognitively healthy populations, previous reports have suggested that higher CRP may have a protective effect (i.e. in slowing decline) once individuals have a dementia diagnosis. This could potentially be via the immune response inflating accordingly, in targeting the vastly accumulated pathology evident in the disease (Lewis and Knight, 2021; Zhang et al., 2023). It is therefore conceivable that the null finding of hs-CRP on cognition in the MCI sample in our study reflects an amalgamation of cases during this intermediate stage between normal ageing and dementia, in which elevated inflammatory reactions may either continue to be detrimental or have started to take on a protective or compensatory role. Thus, MCI may be the critical stage where an appropriate immune response is crucial in determining how the disease may progress. More research is needed in examining factors that influence how the immune response at the MCI stage may affect the progression of disease. Of interest, triggering receptor expressed on myeloid cells 2 (*TREM2*) is a major risk gene for neurodegeneration and AD that plays a key role in changing microglial activity from its homeostatic state to one that is disease-associated (Jay et al., 2017; Krasemann et al., 2017), indicating a potential for variations in microglial reactivity to moderate effects of inflammatory markers on cognition during this stage.

While impairments in executive function and processing speed are common manifestations of white matter disruption, particularly in the periventricular and frontal regions, hs-CRP did not independently predict WMH volumes in our study. This suggests that alternative pathways may underlie the observed relationship between higher hs-CRP and poorer frontal-subcortical neuropsychological functioning. White matter lesions seen on conventional MRI scans represent an advanced stage of cerebrovascular disease and may potentially fail to capture subtle changes (Wersching et al., 2010). Indeed, WMH volumes were significantly correlated with poorer executive function in the MCI group only, but not in the SCD group, which may further indicate that WMHs may not be a sensitive measure in less-impaired populations. As such, it is possible that more precise metrics of white matter disruption may yield greater insights into potential underlying mechanisms. For example, fractional anisotropy obtained from diffusion tensor imaging, or estimates of myelin water fraction may be more sensitive measures of white matter microstructure and network disruption compared to WMHs, and may be useful in early detection of cerebrovascular changes that CRP may be involved in (Boots et al., 2020; Chen et al., 2023; Wersching et al., 2010).

Finally, our exploratory analyses provided preliminary evidence that the relationship between high hs-CRP and cognitive impairment was driven by APOE-ε4 non-carriers only, which could indicate that non-carriers may be more susceptible to the detrimental effects of inflammation on cognitive function. In accordance with this, our findings further suggested that high hs-CRP levels appeared to be linked to better memory performance in APOE-ε4 carriers with MCI. Previous work has also shown that, in older adults experiencing memory complaints, chronically elevated CRP levels are associated with *decreased* cortical Aβ burden, but only in APOE-ε4 carriers (Hooper et al., 2018). While this may suggest that heightened inflammation is beneficial for APOE-ε4 carriers, this may also reflect an over-reactive immune response associated with the APOE-ε4 genotype in these early stages, leading to immune cell exhaustion and homeostatic disruption, and paradoxically contributing to the earlier disease onset and worse prognosis in these individuals (Ferrari-Souza et al., 2023; Millet et al., 2024; Raulin et al., 2022). Nevertheless, the small sample sizes in our exploratory analyses warrants further investigation.

Our clinical findings are based on a well-characterised sample with cognitive data collected using a comprehensive battery of neuropsychological assessments across multiple domains of cognition. This improves upon previous studies where crude measures of global cognition were utilised, which may have lacked sensitivity in detecting effects as well as precluded the ability to delineate neuropsychological functions. Nevertheless, due to the cross-sectional nature of this study, we were unable to investigate the temporal associations of hs-CRP with cognitive decline. Further, hs-CRP concentrations were only measured at a single timepoint. However, hs-CRP levels are observed to fluctuate minimally across multiple measurements (Ockene et al., 2001) and by excluding excessively high concentrations (>10 mg/L) that are likely due to a transitory reaction to infection, hs-CRP levels in this study are presumed to be systemic and an accurate reflection of the chronic state of the innate immune response. Moreover, while we aimed to comprehensively account for well-known modifiable risk factors for dementia, it is possible that other potential confounds were omitted. For example, physical inactivity is known to affect chronic inflammation, and is also a major dementia risk factor (Ribarič, 2022). Finally, the HBA clinic broadly recruits older adults who report experiencing cognitive decline, which is advantageous in generalising our findings to the wider population. However, it is important to note that individuals with SCD and MCI, while considered a cohort at risk of transitioning to dementia, is a heterogeneous group with varying underlying causes for their cognitive impairment that may include, but is not limited to, neurodegenerative mechanisms. To specifically examine SCD/MCI due to neurodegenerative disease, future research examining cohorts of well-characterised participants suggestive to be at the preclinical stages of disease (such as Aβ- and tau-positive individuals) is needed.

### 4.1 Conclusions

The present study contributes to the current body of literature pointing towards variations in the link between hs-CRP and cognition depending on the stage of impairment. Higher hs-CRP concentrations are associated with poorer cognitive performance in cognitively healthy individuals, which we now further understand to include those experiencing SCD, independent of health and lifestyle factors that may contribute to inflammatory processes. However, this association disappears once cognitive impairment becomes clinically evident (as seen in MCI). The harmful relationship of hs-CRP with cognition appears to be specific to neuropsychological domains of executive function and processing speed, though more research is needed to understand the underlying neural mechanisms of these observations. Our findings provide precursory evidence for peripheral inflammation as a potential therapeutic target for cognitive disruption, particularly for APOE-ε4 non-carriers, though further research is needed to better understand the role of various inflammatory processes on disease pathology and progression.

## CRediT authorship contribution statement

**Rachael Yu:** Conceptualization, Formal analysis, Investigation, Methodology, Writing – original draft. **Shawn D. Kong:** Conceptualization, Supervision, Writing – review & editing. **Catriona Ireland:** Data curation. **Genevieve Z. Steiner-Lim:** Supervision, Writing – review & editing. **Kimberley Bassett:** Data curation. **Hannes Almgren:** Data curation, Writing – review & editing. **Dongang Wang:** Data curation. **Chenyu Wang:** Conceptualization. **Johannes C. Michaelian:** Conceptualization, Methodology, Supervision, Writing – review & editing. **Sharon L. Naismith:** Conceptualization, Funding acquisition, Methodology, Supervision, Writing – review & editing.

## Disclosures

The authors report no relevant disclosures.

## Study Funding

This research was supported by 10.13039/501100000925NHMRC Dementia Leadership Fellowship to SLN (APP1135639).

## Declaration of competing interest

The authors declare that they have no known competing financial interests or personal relationships that could have appeared to influence the work reported in this paper.

## Data Availability

The data is not publicly available due to privacy or ethical restrictions.
