# Supplementary material for: Associations of high-sensitivity C-reactive protein with neuropsychological outcomes and cerebral white matter hyperintensities in older adults at risk of dementia
Source: Brain Behav Immun Health. 2024 Dec 11;43:100924. doi: 10.1016/j.bbih.2024.100924 (PMC11728901; doi:10.1016/j.bbih.2024.100924)
Supplement: Multimedia component 1 [file mmc1.docx]

**Supplementary Table S1. Multivariate linear regression analyses of hs-CRP on neuropsychological outcomes, for SCD and MCI groups**

|  | **Model 1** |  | **Model 2** |  |
| --- | --- | --- | --- | --- |
|  | **β [95% CI]** | ***p*** | **β [95% CI]** | ***p*** |
| **Verbal Memory** |  |  |  |  |
| *SCD* |  |  |  |  |
| Hs-CRP (Moderate Risk) | 0.00[-0.18, 0.19] | .954 | 0.06[-0.11, 0.27] | .428 |
| Hs-CRP (High Risk) | 0.01[-0.29, 0.32] | .934 | 0.08[-0.17, 0.49] | .346 |
| **Full model adjusted R^2^** | 11.3% *F*(6, 171) = 4.76 | <.001 | 14.1% *F*(11, 166) = 3.63 | <.001 |
| *MCI* |  |  |  |  |
| Hs-CRP (Moderate Risk) | -0.04[-0.34, 0.17] | .522 | -0.02[-0.30, 0.22] | .776 |
| Hs-CRP (High Risk) | 0.11[-0.34, 0.41] | .855 | 0.04[-0.28, 0.52] | .565 |
| **Full model adjusted *R*^2^** | 5.4% *F*(6, 276) = 3.67 | .002 | 6.7%  *F*(11, 271) = 2.84 | .002 |
| **Executive Function** |  |  |  |  |
| *SCD* |  |  |  |  |
| Hs-CRP (Moderate Risk) | -0.02[-0.20, 0.16] | **.**825 | -0.01[-0.19, 0.17] | .914 |
| Hs-CRP (High Risk) | -0.20[-0.62, -0.05] | .022 | -0.20[-0.65, -0.04] | .025 |
| **Full model adjusted *R*^2^** | 5.3% *F*(6, 152) = 2.49 | .025 | 12.1% *F*(11, 147) = 2.97 | .001 |
| *MCI* |  |  |  |  |
| Hs-CRP (Moderate Risk) | -0.08[-0.29, 0.06] | .206 | -0.04[-0.24, 0.12] | .499 |
| Hs-CRP (High Risk) | 0.00[-0.26, 0.25] | .964 | 0.04[-0.20, 0.35] | .595 |
| **Full model adjusted *R*^2^** | 4.9% *F*(6, 261) = 3.30 | .004 | 7.9%  *F*(11, 256) = 3.09 | <.001 |
| **Processing Speed** |  |  |  |  |
| *SCD* |  |  |  |  |
| Hs-CRP (Moderate Risk) | -0.02[-0.17, 0.13] | .767 | -0.06[-0.21, 0.09] | .446 |
| Hs-CRP (High Risk) | -0.10[-0.38, 0.10] | .257 | -0.19[-0.53, 0.00] | .048 |
| **Full model adjusted *R*^2^** | 2.7% *F*(6, 156) = 1.74 | .115 | 5.6%  *F*(11, 151) = 1.88 | .047 |
| *MCI* |  |  |  |  |
| Hs-CRP (Moderate Risk) | -0.03[-0.24, 0.14] | .604 | -0.01[-0.25, 0.17] | .838 |
| Hs-CRP (High Risk) | 0.01[-0.25, 0.30] | .871 | 0.02[-0.25, 0.35] | .727 |
| **Full model adjusted *R*^2^** | 0.9% *F*(6, 270) = 1.43 | .203 | 1.3%  *F*(11, 265) = 1.34 | .205 |
|  |  |  |  |  |

Note. Moderate- and high-risk CRP groups were compared to the referent group, low-risk hs-CRP. β = standardised beta coefficient; CI = confidence interval; SCD = subjective cognitive decline; MCI = mild cognitive impairment; Hs-CRP = high-sensitivity C-reactive protein.

Model 1 adjusted for age, sex, years of education and total disease burden (CIRS-G).

Model 2 additionally adjusted for depressive symptoms (GDS-15), alcohol consumption, BMI, cardiovascular health (FGCRS) and sleep quality (PSQI total score).

**Supplementary Table S2. Multivariate linear regression analyses of hs-CRP on WMH volumes, for SCD and MCI groups**

|  | **Model 1** |  | **Model 2** |  |
| --- | --- | --- | --- | --- |
|  | **β (95% CI)** | ***p*** | **β (95% CI)** | ***p*** |
| **Total WMH volume** |  |  |  |  |
| *SCD* |  |  |  |  |
| Hs-CRP (Moderate Risk) | 0.11[-1.13, 0.16] | .198 | 0.12[-0.19, 0.94] | .192 |
| Hs-CRP (High Risk) | 0.02[-0.18, 0.88] | .855 | -0.04[-1.24, 0.86] | .719 |
| **Full model adjusted R^2^** | 34.9% *F*(8, 100) = 8.25 | <.001 | 36.0%  *F*(13, 95) = 5.68 | <.001 |
| *MCI* |  |  |  |  |
| Hs-CRP (Moderate Risk) | -0.04[-0.62, 0.32] | .517 | -0.04[-0.64, 0.34] | .546 |
| Hs-CRP (High Risk) | -0.08[-1.09, 0.29] | .254 | -0.07[-1.11, 0.44] | .389 |
| **Full model adjusted R^2^** | 32.9%  *F*(8, 162) = 11.41 | <.001 | 34.3%  *F*(13, 157) = 7.83 | <.001 |
| **Periventricular** |  |  |  |  |
| *SCD* |  |  |  |  |
| Hs-CRP (Moderate Risk) | 0.14[-0.12, 1.51] | .095 | 0.16[-0.12, 1.67] | .088 |
| Hs-CRP (High Risk) | 0.08[-0.77, 2.05] | .369 | 0.06[-1.13, 2.17] | .532 |
| **Full model adjusted R^2^** | 34.2% *F*(8, 100) = 8.01 | <.001 | 32.4%  *F*(13, 95) = 4.98 | <.001 |
| *MCI* |  |  |  |  |
| Hs-CRP (Moderate Risk) | -0.05[-1.01, 0.44] | .438 | -0.03[-0.93, 0.55] | .611 |
| Hs-CRP (High Risk) | -0.04[-1.34, 0.73] | .533 | -0.00[-1.21, 1.15] | .965 |
| **Full model adjusted R^2^** | 40.5% *F*(8, 162) = 15.48 | <.001 | 42.8%  *F*(13, 157) = 10.77 | <.001 |
| **Deep white matter** |  |  |  |  |
| *SCD* |  |  |  |  |
| Hs-CRP (Moderate Risk) | 0.11[-0.21, 0.81] | .241 | 0.13[-0.20, 0.88] | .218 |
| Hs-CRP (High Risk) | 0.11[-0.40, 1.34] | .285 | 0.07[-0.67, 1.34] | .514 |
| **Full model adjusted R^2^** | 14.7% *F*(8, 100) = 3.33 | .002 | 15.2%  *F*(13, 95) = 2.49 | .006 |
| *MCI* |  |  |  |  |
| Hs-CRP (Moderate Risk) | -0.13[-0.72, 0.05] | .088 | -0.15[-0.79, 0.02] | .061 |
| Hs-CRP (High Risk) | -0.12[-1.02, 0.10] | .109 | -0.15[-1.19, 0.10] | .096 |
| **Full model adjusted R^2^** | 13.7% *F*(8, 162) = 4.38 | <.001 | 13.3%  *F*(13, 157) = 3.00 | <.001 |
| **Frontal lobe** |  |  |  |  |
| *SCD* |  |  |  |  |
| Hs-CRP (Moderate Risk) | 0.11[-0.24, 1.12] | .201 | 0.13[-0.22, 1.22] | .166 |
| Hs-CRP (High Risk) | 0.01[-1.10, 1.24] | .904 | -0.03[-1.54, 1.32] | .835 |
| **Full model adjusted R^2^** | 30.1%  *F*(8, 100) = 6.82 | <.001 | 31.2%  *F*(13, 95) = 4.76 | <.001 |
| *MCI* |  |  |  |  |
| Hs-CRP (Moderate Risk) | -0.02[-0.64, 0.48] | .767 | -0.03[-0.72, 0.45] | .646 |
| Hs-CRP (High Risk) | -0.04[-1.05, 0.59] | .583 | -0.06[-1.29, 0.57] | .446 |
| **Full model adjusted R^2^** | 29.6%  *F*(8, 162) = 9.95 | <.001 | 29.9%  *F*(13, 157) = 6.58 | <.001 |
|  |  |  |  |  |

Note. Moderate- and high-risk CRP groups were compared to the referent group, low-risk hs-CRP. All WMH volumes were log-transformed. β = standardised beta coefficient; CI = confidence interval; WMH = white matter hyperintensities; SCD = subjective cognitive decline; MCI = mild cognitive impairment; Hs-CRP = high-sensitivity C-reactive protein.

Model 1 adjusted for age, sex, years of education, total disease burden (CIRS-G) and scan type.

Model 2 additionally adjusted for depressive symptoms (GDS-15), alcohol consumption, BMI, cardiovascular health (FGCRS) and sleep quality (PSQI total score).
